# Girls with a History of Premature Adrenarche Have Advanced Growth and Pubertal Development at the Age of 12 Years

**DOI:** 10.3389/fendo.2017.00291

**Published:** 2017-10-31

**Authors:** Jani Liimatta, Pauliina Utriainen, Raimo Voutilainen, Jarmo Jääskeläinen

**Affiliations:** ^1^Department of Pediatrics, University of Eastern Finland and Kuopio University Hospital, Kuopio, Finland

**Keywords:** premature adrenarche, dehydroepiandrosterone sulfate, insulin-like growth factor I, growth, puberty, menarche

## Abstract

**Background:**

Premature adrenarche (PA) has been linked to early thelarche and menarche, but longitudinal data on growth and pubertal development after PA are insufficient.

**Methods:**

Growth and pubertal development of mostly full-term and appropriate for gestational age-born 43 PA (36 girls) and 63 control children (52 girls) were analyzed prospectively. Children examined first at the mean age of 7.6 years were reexamined at the mean age of 12.0 years.

**Results:**

The PA girls but not the boys were taller and had higher body mass index (BMI) than the controls. A higher proportion of the PA than control girls had reached menarche, while the same percentage of the PA and control boys were at Tanner genital stage ≥2. The PA girls with premature pubarche (PP) were taller but not heavier and had more often reached menarche by the age of 12 years than the PA girls without PP. The PA girls with menarche had lower birth length (BL) and higher prepubertal insulin-like growth factor 1 (IGF-1) concentrations compared with non-menarcheal PA girls. In logistic regression analyses for all girls, lower BL standard deviation score, earlier maternal menarche, and higher prepubertal IGF-1 were independently associated with menarche.

**Conclusion:**

At 12 years of age, the PA girls had higher BMI, advanced linear growth, and accelerated pubertal development with earlier menarche than the control girls. The PA girls with PP were taller and had earlier menarche than the PA girls without PP. Lower BL and higher prepubertal IGF-1 concentration were predictive factors for menarche by the age of 12 years.

## Introduction

In adrenarche, developing adrenocortical zona reticularis produces increasing amounts of adrenal androgen precursors, mostly dehydroepiandrosterone sulfate (DHEAS). Increasing androgenic activity leads to clinical signs: adult-type body odor, oily hair and skin, comedones/acne, and axillary and pubic hair. If the clinical signs, together with elevated DHEAS concentrations, are seen before the age of 8 years in a girl or 9 years in a boy, adrenarche is regarded as premature [reviewed in Ref. (1, 2)]. In Finland, the prevalence of premature adrenarche (PA) is 8.6% in girls and 1.8% in boys (3). Despite a relatively high prevalence, the long-term outcome of PA is still poorly known.

In several studies, PA has been linked to unfavorable metabolic features including obesity (4, 5) and hyperinsulinism or insulin resistance (6–8). Low birth weight or being born small for gestational age (SGA) has been associated with PA in retrospective studies from Australia, Brazil, and Spain (5, 9, 10). Advanced prepubertal growth in height has been reported in most PA cohort studies (11–14). One possible link between accelerated statural growth and PA is insulin-like growth factor 1 (IGF-1) (15) whose increased concentrations have been found in PA children (14, 16, 17).

Only few PA cohorts have been followed intensively until adolescence or adulthood (9, 18). In these longitudinal studies, PA girls have had earlier timing of thelarche and menarche than their peers. Of note, a substantial percentage of the PA girls in these studies have been born SGA, which itself may lead to early catch-up growth and earlier menarche (19). Earlier timing of menarche was seen also in one retrospective study (13). Although children with PA usually have advanced bone age (11, 17) and prepubertal statural growth (11–14), final height is mostly reported to be normal (9, 12, 13, 18).

At the baseline of our longitudinal follow-up study on PA, overweight, hyperinsulinism, decreased insulin sensitivity, and childhood metabolic syndrome were more common in the PA than control girls at the mean age of 7.6 years (6). The girls with PA were also taller than their peers with higher serum IGF-1 concentrations (14). In this follow-up study, we examined whether the anthropometric differences found in prepuberty persist until adolescence and if PA is associated with advanced pubertal development at 12 years of age. Finally, we explored which prepubertal factors predict earlier menarche in PA and healthy girls.

## Subjects and Methods

### Subjects and Design

Our original cohort study on 73 Finnish children with PA and 99 age- and sex-matched healthy controls has been reported previously (20). We invited the children to attend a follow-up visit at the age of 12 years. Altogether 106 subjects participated (61.6% of the original cohort; 36 PA, 52 control girls; 7 PA, 11 control boys), and they were all examined at the pediatric outpatient clinic of Kuopio University Hospital. The PA children had presented with at least one clinical sign of adrenal androgen action before the age of 8/9 years (girls/boys, respectively) together with a serum DHEAS concentration over 1 µmol/L and other reasons of hyperandrogenism (including central puberty, congenital adrenal hyperplasia, androgen producing tumor) were excluded. Five children had been born SGA [birth weight <−2 standard deviation score (SDS); 4.7% overall; 1/36 PA vs 3/52 control girls; 1/7 PA vs 0/11 control boys; both NS]. Five girls, but none of the boys had been born preterm (4.7% overall; 1/36 PA vs 4/52 control girls; NS). The study protocol was approved by the Research Ethics Committee of the Hospital District of Northern Savo, and an informed consent was obtained from the children and their parents in accordance with the ethical principles stated in the Declaration of Helsinki.

### Clinical Evaluation

Gestational age, birth size, anthropometric measures, pubertal stage, and biochemical results at the mean age of 7.6 years had been recorded earlier. At the current 12-year-age follow-up visit, anthropometric measures, pubertal stage, and menarcheal timing were assessed. Bone age determination was not included in the study protocol for ethical reasons (i.e., radiation exposure). The parents were enquired upon their heights and timing of puberty (maternal age at menarche and paternal age during pubertal peak height velocity). These were subjective estimates given by the parents, but as both growth and puberty are systematically monitored in Finland until the end of secondary education, individuals are well aware of their final height and pubertal tempo. Paternal pubertal growth spurt was classified into three categories (early, on-time, and late) as compared with that of their peers: a difference more than a year was considered as early or late timing of the pubertal growth spurt.

Height was measured with a calibrated Harpenden stadiometer (Holtain Ltd., Crymych, UK) and recorded to the nearest 0.1 cm as the mean of three repeated measurements. Weight was measured with a calibrated electronic scale after an overnight fast and recorded to the nearest 0.1 kg. Waist circumference was measured after expiration at mid-distance between the bottom of the rib cage and the top of the iliac crest. Body mass index (BMI) was calculated as the weight in kilograms divided by the square of height in meters. Birth length (BL), birth weight, height, and BMI SDS were calculated with current Finnish growth references (21, 22). Pubertal development was evaluated by a trained physician (P.U.) with Tanner staging scores (23, 24). Age of menarche was defined as precisely as possible by interviewing the girls and their parents. Corrected mid-parental height (cMPH) was calculated by the method of Tanner (25): mid-parental height − 6.5 cm for girls and mid-parental height + 6.5 cm for boys.

### Biochemical Analyses

All biochemical samples had been drawn and analyzed at the baseline visit (mean age of 7.6 years) and the analytical methods have been described more closely in the previous reports (6, 14, 20). The blood samples were drawn after an overnight fast and stored at −80°C until assayed. Serum insulin concentrations were analyzed with a specific time-resolved fluoroimmunoassay by AutoDelfia (PerkinElmer Life and Analytical Sciences Wallac Oy, Turku, Finland), DHEAS, and androstenedione concentrations with specific Coat-A-Count RIAs (Diagnostic Products Corporation, Los Angeles, CA, USA), and IGF-1 concentration by an immunochemiluminometric assay using a Diagnostic Products IMMULITE 2000 analyzer.

### Statistical Analyses

All statistical analyses were performed using the SPSS 23.0 software (IBM Corp., Armonk, NY, USA). The distributions for normality were first tested with the Shapiro–Wilk test and visually from the histograms. The independent samples *t*-test for normally distributed variables and the Mann–Whitney *U* test for non-normally distributed variables were used to compare differences in the means and medians between the study groups, respectively. To evaluate statistical differences in pubertal development between the study groups in crosstab settings, the Pearson χ^2^ test or Fisher’s exact test was used. Binary logistic regression analyses were used to evaluate predictive factors for menarche (categorical dependent variable: menarche, yes or no). Continuous non-normally distributed biochemical covariates in the regression analyses were first logarithmically transformed and then used as SDS. The associations or statistical differences with *P* < 0.05 were considered significant.

## Results

Results were analyzed separately for females and males. Characteristics of the PA and control girls and boys at birth, at the age of 7 years (=baseline), and at the age of 12 years (=follow-up visit) are depicted in Table 1. Subgroup comparisons of the PA girls with (PA + PP) and without premature pubarche (PA − PP) at the baseline, and the PA girls with and without menarche at the age of 12 years are depicted in Table 2.

**Table 1:** Characteristics of children with PA and control children at birth, at the age of 7 years, and at the age of 12 years.

	Girls			Boys		
	PA, *n* = 36	Control, *n* = 52	*P*	PA, *n* = 7	Control, *n* = 11	*P*
**At birth**						
Gestational age, weeks	40.4 (38.9 to 41.0)	40.1 (39.3 to 41.1)	0.905^a^	39.0 (38.3 to 41.0)	40.6 (39.0 to 41.4)	0.496^a^
Birth weight, kg	3.47 (3.11 to 3.72)	3.54 (3.27 to 3.92)	0.293^a^	3.59 (2.75 to 4.02)	3.85 (3.41 to 4.01)	0.258^a^
Birth weight SDS	−0.12 (−0.51 to 0.27)	0.05 (−0.25 to 0.34)	0.486	−0.30 (−1.52 to 0.91)	0.31 (−0.33 to 0.96)	0.267
Birth length (BL), cm	50.0 (48.0 to 51.0)	51.0 (50.0 to 52.0)	**0.037**^a^	51.5 (47.0 to 53.0)	52.0 (50.0 to 52.0)	0.644^a^
BL SDS	−0.11 (−0.45 to 0.24)	0.27 (−0.02 to 0.57)	0.073	0.02 (−1.57 to 1.60)	0.36 (−0.39 to 1.11)	0.612

**At 7 years of age**						
Age, years	7.6 (7.2 to 8.1)	7.6 (7.1 to 8.0)	0.877^a^	7.3 (6.9 to 8.9)	8.4 (7.2 to 8.5)	0.892^a^
Height SDS	1.02 (0.64 to 1.40)	−0.16 (−0.46 to 0.14)	**<0.001**	0.49 (−0.85 to 1.82)	0.42 (−0.21 to 1.04)	0.902
BMI SDS	1.04 (0.65 to 1.43)	0.17 (−0.12 to 0.46)	**<0.001**	1.11 (0.07 to 2.14)	0.75 (0.00 to 1.50)	0.520
DHEAS, μmol/L	2.1 (1.5 to 2.9)	0.8 (0.5 to 1.3)	**<0.001**^a^	1.6 (1.3 to 4.3)	0.7 (0.5 to 2.7)	0.188^a^
Androstenedione, nmol/L	3.2 (2.1 to 3.9)	1.6 (1.9 to 2.2)	**<0.001**^a^	2.4 (2.2 to 3.0)	1.2 (0.5 to 2.4)	0.051^a^
Insulin, mU/L	5.8 (4.3 to 7.4)	4.1 (3.2 to 5.3)	**0.006**^a^	4.7 (2.2 to 7.9)	4.7 (3.2 to 6.6)	1^a^
IGF-1, nmol/L	23.9 (20.0 to 30.5)	19.0 (16.0 to 23.0)	**0.001**^a^	23.0 (14.0 to 29.0)	20.0 (13.0 to 23.0)	0.439^a^

**At 12 years of age**						
Age, years	12.0 (12.0 to 12.1)	12.0 (12.0 to 12.1)	0.420^a^	12.2 (11.9 to 12.3)	12.0 (11.9 to 12.1)	0.134^a^
Height, cm	160.0 (157.1 to 162.8)	153.3 (151.4 to 155.2)	**<0.001**	154.9 (142.9 to 166.9)	153.8 (149.7 to 157.9)	0.808
Height SDS	0.88 (0.48 to 1.28)	−0.08 (−0.35 to 0.20)	**<0.001**	0.19 (−1.46 to 1.84)	0.16 (−0.44 to 0.77)	0.965
Sitting height-to-height ratio	0.53 (0.52 to 0.53)	0.52 (0.52 to 0.53)	0.122	0.52 (0.51 to 0.53)	0.52 (0.51 to 0.53)	0.699
Weight, kg	53.9 (45.5 to 67.3)	43.4 (37.3 to 51.0)	**<0.001**^a^	54.9 (39.1 to 64.0)	47.7 (36.8 to 66.1)	0.821^a^
BMI, kg/m^2^	20.6 (17.9 to 25.7)	18.5 (16.6 to 21.3)	**0.002**^a^	20.0 (17.6 to 26.7)	19.7 (17.1 to 25.7)	0.618^a^
BMI SDS	0.81 (0.49 to 1.14)	0.13 (−0.15 to 0.41)	**0.002**	0.92 (−0.23 to 2.06)	0.66 (−0.12 to 1.44)	0.662
Waist-to-height ratio	0.42 (0.38 to 0.47)	0.41 (0.38 to 0.45)	0.235^a^	0.45 (0.43 to 0.59)	0.45 (0.41 to 0.54)	0.649^a^
cMPH, cm	166.9 (165.1 to 168.8)	165.0 (163.7 to 166.2)	0.064	175.6 (169.7 to 181.5)	177.2 (174.6 to 179.9)	0.514
Maternal age at menarche, years	12.7 (11.1 to 14.0)	13.0 (12.0 to 13.0)	0.238^a^	13.0 (12.0 to 14.0)	13.0 (12.0 to 13.5)	0.547^a^
Early paternal pubertal growth spurt, yes %	22	6	**0.024**^b^	0	0	1^c^

*Values are expressed as mean (95% confidence interval), and statistical differences between the study groups were analyzed using independent samples *t*-test, unless noted otherwise. Significant *p*-values (<0.05) are highlighted with bold*.

*^a^Median (interquartile range) and Mann–Whitney *U* test for non-normally distributed variables*.

*^b,c^Percentage and Pearson χ^2^ test^b^ or Fisher’s exact test^c^ for categorical variable*.

*PA, premature adrenarche; SDS, standard deviation score; BMI, body mass index; DHEAS, dehydroepiandrosterone sulfate; IGF-1, insulin-like growth factor 1; cMPH, corrected mid-parental height*.

**Table 2:** Comparisons of the follow-up data between the subgroups of the PA girls with (PA + PP) and without premature pubarche (PA − PP), and the PA girls with (PA + M) and without menarche (PA − M) at the age of 12 years.

	PA girls					
	PA + PP, *n* = 20	PA − PP, *n* = 16	*P*	PA + M, *n* = 13	PA − M, *n* = 23	*P*
**At birth**						
Gestational age, weeks	40.2 (38.9 to 41.0)	40.4 (39.0 to 41.0)	0.949^a^	40.3 (38.9 to 41.0)	40.4 (39.2 to 41.4)	0.882^a^
Birth weight, kg	3.44 (3.01 to 3.72)	3.52 (3.13 to 3.80)	0.787^a^	3.41 (3.11 to 3.57)	3.63 (3.19 to 4.01)	0.122^a^
Birth weight SDS	−0.16 (−0.75 to 0.43)	−0.07 (−0.63 to 0.49)	0.808	−0.36 (−0.71 to −0.01)	0.30 (−0.62 to 1.23)	0.097
Birth length (BL), cm	49.8 (48.0 to 50.8)	50.0 (48.3 to 51.0)	0.498^a^	49.0 (48.0 to 50.0)	51.0 (49.5 to 51.8)	**0.028**^a^
BL SDS	−0.15 (−0.62 to 0.32)	−0.06 (−0.63 to 0.52)	0.785	−0.45 (−0.77 to −0.13)	0.50 (−0.22 to 1.22)	**0.005**

**At 7 years of age**						
Height SDS	1.34 (0.78 to 1.90)	0.61 (0.14 to 1.09)	0.051	1.23 (0.80 to 1.66)	0.63 (−0.14 to 1.40)	0.125
BMI SDS	1.23 (0.74 to 1.72)	0.81 (0.14 to 1.47)	0.274	1.20 (0.75 to 1.65)	0.77 (−0.02 to 1.57)	0.289
DHEAS, μmol/L	2.5 (1.7 to 3.6)	1.8 (1.4 to 2.2)	**0.037**^a^	2.1 (1.6 to 3.1)	2.1 (1.4 to 2.5)	0.830^a^
Androstenedione, nmol/L	3.3 (2.7 to 4.4)	2.4 (1.3 to 3.4)	**0.037**^a^	3.1 (2.7 to 4.0)	3.2 (1.6 to 4.4)	0.564^a^
Insulin, mU/L	6.0 (5.5 to 7.8)	4.5 (3.1 to 7.4)	0.161^a^	6.1 (4.7 to 7.4)	4.5 (2.7 to 7.1)	0.121^a^
IGF-1, nmol/L	26.0 (22.2 to 31.0)	21.0 (17.3 to 26.0)	**0.037**^a^	26.0 (21.8 to 33.8)	20.0 (17.5 to 24.0)	**0.009**^a^

**At 12 years of age**						
Height, cm	162.6 (158.9 to 166.2)	156.9 (152.5 to 161.3)	**0.032**	162.5 (159.7 to 165.2)	155.8 (149.8 to 161.7)	**0.036**
Height SDS	1.25 (0.73 to 1.76)	0.44 (−0.18 to 1.07)	**0.041**	1.23 (0.84 to 1.63)	0.28 (−0.55 to 1.11)	**0.039**
Weight, kg	55.5 (46.2 to 66.6)	49.9 (39.5 to 70.2)	0.301^a^	55.6 (48.0 to 67.4)	45.7 (38.3 to 68.9)	0.078^a^
BMI, kg/m^2^	20.8 (18.8 to 25.3)	20.1 (17.5 to 26.5)	0.702^a^	21.4 (19.0 to 25.4)	18.1 (17.6 to 26.1)	0.249^a^
BMI SDS	0.87 (0.47 to 1.27)	0.74 (0.16 to 1.32)	0.692	0.96 (0.58 to 1.34)	0.56 (−0.08 to 1.20)	0.227
cMPH, cm	167.0 (164.2 to 169.7)	166.9 (164.6 to 169.2)	0.987	166.8 (164.6 to 169.1)	167.2 (163.9 to 170.5)	0.842
Maternal age at menarche, years	12.9 (11.1 to 14.0)	12.0 (11.3 to 13.8)	0.759^a^	12.0 (11.0 to 13.0)	13.0 (12.3 to 14.0)	**0.012**^a^
Early paternal pubertal growth spurt, yes %	30	13	0.209^b^	26	15	0.458^b^

*Values are expressed as mean (95% confidence interval), and statistical differences between the study groups are analyzed using independent samples *t*-test, unless noted otherwise. Significant *p*-values (<0.05) are highlighted with bold*.

*^a^Median (interquartile range) and Mann–Whitney *U* test for non-normally distributed variables*.

*^b^Percentage and Pearson χ^2^ test for categorical variable*.

*PA, premature adrenarche; PP, premature pubarche; M, menarche; SDS, standard deviation score; BMI, body mass index; DHEAS, dehydroepiandrosterone sulfate; IGF-1, insulin-like growth factor 1; cMPH, corrected mid-parental height*.

### Anthropometry

Gestational age, birth weight, and age at baseline or follow-up visit did not differ between the PA and control girls (Table 1). The PA girls participating in the current examination at the age of 12 years had significantly lower BL than the control girls, but the difference in BL SDS did not reach statistical significance (Table 1). At the follow-up visit, the girls with a history of PA were taller (height SDS 0.88 vs −0.08, *P* < 0.001) and had higher BMI SDS (0.81 vs 0.13, *P* = 0.002) than the control girls (Table 1). Moreover, the PA + PP girls were taller (height SDS 1.25 vs 0.44, *P* = 0.041) but not heavier than the PA − PP girls at the age of 12 years (Table 2). No statistically significant differences were detected in anthropometrics between the PA and control boys.

### Pubertal Development

**Figure 1:**
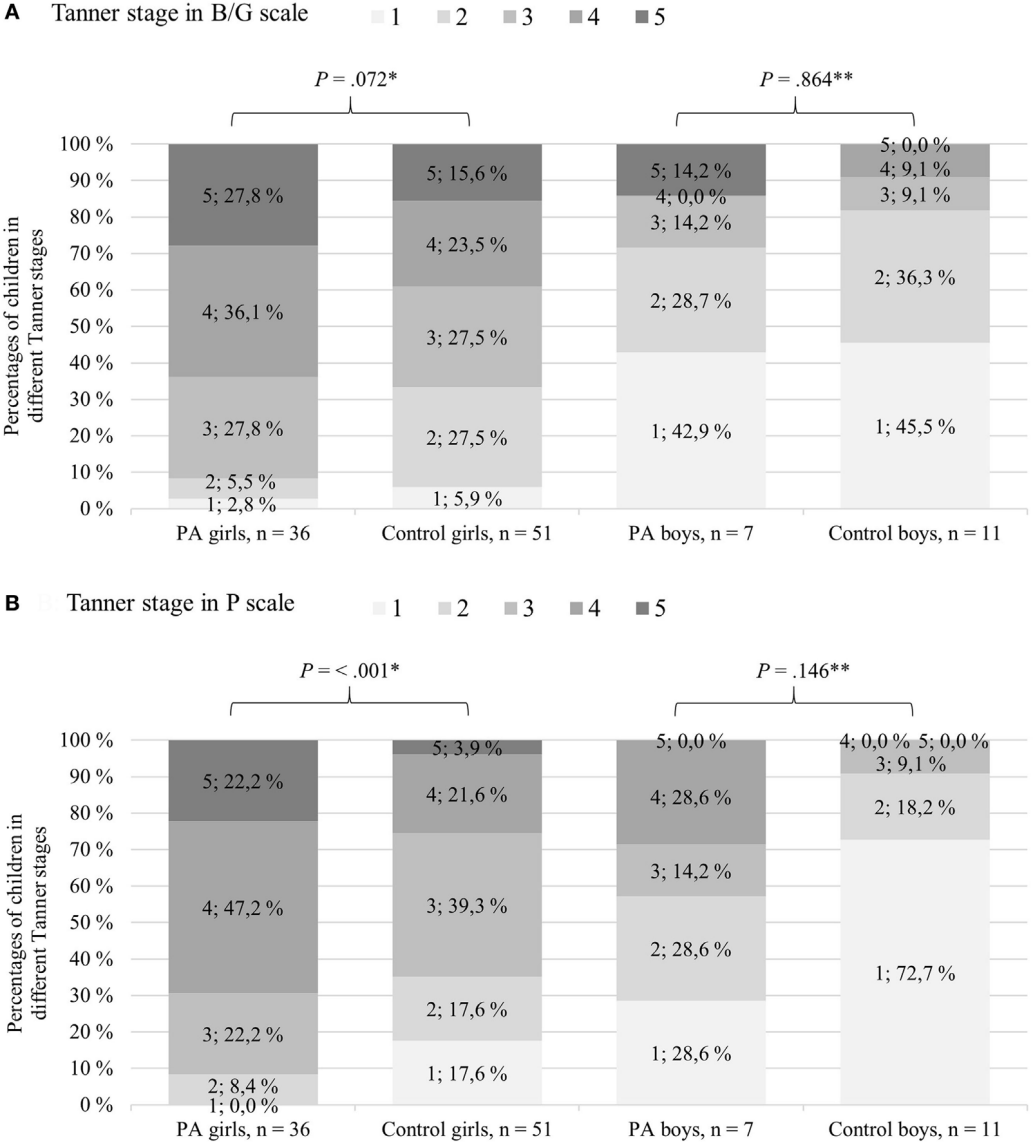
Pubertal development in Tanner B/G **(A)** and P scale **(B)** for children with premature adrenarche (PA) and controls at the age of 12 years. Gray shades represent Tanner stage scores, and darker shades reflect more advanced pubertal development. *Pearson χ^2^ test or **Fisher’s exact test was used to analyze statistical differences between the PA and control girls/boys, respectively.

Pubertal development of the children with PA and controls is depicted in Figure 1 and Table 3. There were no significant differences in the percentage of children with ongoing puberty (Tanner B ≥ 2 or testicular volume ≥ 4 mL) between the PA and control children; 95.4% of the participating girls and 55.6% of the boys were pubertal. However, in a more detailed analysis of pubertal development, the PA girls were more advanced than the controls as they had more often reached menarche (63.9 vs 25.0%, *P* < 0.001; Table 3), had higher pubic hair stage (*P* < 0.001; Figure 1), and a trend for more advanced breast development (*P* = 0.072; Figure 1). When analyzing the PA girls, the PA + PP girls had higher percentage of reached menarche compared with that of the PA − PP girls (80.0 vs 43.8%, *P* = 0.024; Table 3). Among the boys, no statistical difference in pubertal development was found between the PA and control groups.

**Table 3:** Pubertal development of children with PA and control children at the age of 12 years, including subgroup analyses between the PA girls with (PA + PP) and without premature pubarche (PA − PP).

	All girls*			PA girls*			All boys**		
	PA, *n* = 36	Control, *n* = 52	*P*	PA + PP, *n* = 20	PA − PP, *n* = 16	*P*	PA, *n* = 7	Control, *n* = 11	*P*
Thelarche or testicular enlargement, yes^a^	35 (97.2%)	48 (94.1%)	0.496	20 (100%)	15 (93.8%)	0.257	4 (57.1%)	6 (54.5%)	1
Pubarche, yes^b^	36 (100%)	42 (82.4%)	**0.008**	20 (100%)	16 (100%)	1	5 (71.4%)	3 (27.3%)	0.145
Menarche, yes	23 (63.9%)	13 (25.0%)	**<0.001**	16 (80.0%)	7 (43.8%)	**0.024**	Not applicable		

Values are presented as *n* (%), and statistical differences between the study groups were analyzed using either *Pearson χ^2^ test or **Fisher’s exact test. Significant *p*-values (<0.05) are highlighted with bold.

*^a^Tanner B ≥ 2 or testicular volume ≥ 4 mL*.

*^b^Tanner P ≥ 2*.

*Both ^a^ and ^b^ n = 51 for control girls*.

*PA, premature adrenarche; PP, premature pubarche*.

### Associations with Menarcheal Timing

Logistic regression analyses on determinants of menarche for all girls is shown in Table 4. In univariate analyses, lower BL SDS, the history of PA, earlier maternal menarche, higher prepubertal BMI, serum IGF-1, and DHEAS concentrations were all significant determinants of earlier menarche (data not shown). In multivariate analyses, lower BL SDS, lower maternal age at menarche, and a higher prepubertal IGF-1 concentration were independently associated with earlier menarche. In subgroup analyses comparing the PA girls with and without menarche, the postmenarcheal PA girls had lower BL, higher prepubertal IGF-1 concentrations, and taller stature at the age of 12 years (Table 2).

**Table 4:** Logistic regression models predicting menarche by the age of 12 years.

	Regression coefficient^a^	*P*	Odds ratio (95% CI)^a^
**Model 1**			
Age, years^b^	0.51	0.292	1.66 (0.65–4.25)
Birth length SDS	−2.16	**0.002**	0.12 (0.03–0.46)
Birth weight SDS	0.59	0.286	1.81 (0.61–5.35)
PA	1.21	0.083	3.37 (0.85–13.3)
Prepubertal BMI SDS	0.66	0.052	1.94 (1.00–3.78)
Maternal age at menarche, years	−1.19	**0.001**	0.30 (0.15–0.62)
Early paternal pubertal growth spurt^c^	1.10	0.302	2.99 (0.37–23.9)
**Model 2**			
Age, years^b^	0.14	0.718	1.14 (0.55–2.38)
DHEAS SDS	0.20	0.625	1.22 (0.55–2.70)
Androstenedione SDS	0.32	0.478	1.38 (0.57–3.35)
Insulin SDS	0.30	0.291	1.35 (0.77–2.35)
IGF-1 SDS	0.76	**0.013**	2.14 (1.18–3.88)

*Dependent variable: menarche at the age of 12 years, yes or no*.

*Estimated size of the effects of all variables in the models: Model 1, *R*^2^ = 0.65; Model 2, *R*^2^ = 0.30. Significant *p*-values (<0.05) are highlighted with bold*.

*^a^Regression coefficients and odds ratios are adjusted for all other variables in the models*.

*^b^Age at the initial evaluation visit in prepuberty*.

*^c^Paternal pubertal growth spurt over 1 year earlier compared with their peers*.

*CI, confidence interval; SDS, standard deviation score; PA, premature adrenarche; BMI, body mass index; DHEAS, dehydroepiandrosterone sulfate; IGF-1, insulin-like growth factor 1*.

### Comparison of Parental Heights and Pubertal Timing between the Study Groups

The history of parental pubertal development and cMPH are depicted in Tables 1 and 2. cMPH had a trend to be higher in the PA girls compared with the control ones (166.9 vs 165.0 cm, *P* = 0.064). Maternal age at menarche did not differ between the PA and control girls. However, maternal median age at menarche was lower in the PA girls with menarche compared with those without menarche (12.0 vs 13.0 years, *P* = 0.012). Percentages of the fathers with early pubertal growth spurt was higher in the PA girls compared with the control girls (22 vs 6%, *P* = 0.024). No differences were found in cMPH, maternal age at menarche, or the percentage of the fathers with early pubertal growth spurt between the PA and control boys or the PA + PP and PA − PP girls (Tables 1 and 2).

## Discussion

In this follow-up study, we examined mostly full-term and appropriate for gestational age (AGA)-born children with a history of PA and their healthy controls at the age of 12 years. In prepuberty, the PA girls had presented with advanced statural growth, higher weight-for-height, and higher serum IGF-1 concentrations than the control children (14). Most of these children (61.6%) participated in this follow-up visit and, compared with healthy peers, the PA girls were still taller and heavier, and had more advanced pubertal development. Moreover, those PA girls who had presented with premature pubarche (PP) at the initial visit in prepuberty were taller and had higher percentage of reached menarche at the age of 12 years than the PA girls without PP. The number of boys in this study was too small to detect any statistically significant differences in anthropometry or pubertal development. In logistic regression analyses, earlier maternal menarche, lower BL SDS, and higher serum IGF-1 concentrations were significant explanatory factors for earlier menarche in all girls of this study. The PA girls with menarche had lower BL SDS, higher prepubertal IGF-1 concentrations and taller stature at the age of 12 years than the PA girls without menarche.

To date, there are only two published reports investigating PA children in a prospective setting (9, 18). In both of these, contrary to our study, there was no control group, many participants were born SGA, and all children with PA had PP. In one of these studies, 187 girls with PP were followed from diagnosis to early adulthood: the PP children had earlier menarche than the girls in the general population (18). In the other more recent longitudinal study, 52 girls with PP were followed from the diagnosis of PP (9). Approximately one-third of these girls were obese, which was more than in the reference population. Both thelarche and menarche occurred earlier than in the general population being still within the normal range. One-third (30.8%) of these children had reached final height, with no difference to the normal population standards.

Some cross-sectional studies have explored pubertal development in children with a history of PA/PP. In a Finnish study investigating growth and maturation of 23 teenaged mostly AGA-born PA girls, menarche occurred 0.5 years earlier than in the general female population (13). Evidence for earlier pubertal timing in PA children was also reported in two other retrospective studies on 85 AGA-born Israeli and 37 multiethnic PA girls: menarche occurred earlier in PA girls than controls (26) or reference population (27), and thelarche occurred earlier compared with the control group (27). In contrast, some authors did not find any difference in pubertal timing in PA girls compared with general population (12). Our present findings are well in line with these previous longitudinal and retrospective studies showing slightly earlier pubertal development after PA.

There are also some retrospective or cross-sectional studies on pubertal growth and body composition in subjects with the history of PA. In one study, non-obese postmenarcheal adolescent girls with the history of PP had higher total body fat mass and more unfavorable fat distribution than those without a history of PP (28). In two other studies, PA children were taller than the general population at early puberty (12, 13). Our study, however, is the first one comparing pubertal height between PA/PP children and carefully matched controls in a follow-up setting, and confirms that PA girls remain taller still at pubertal age.

In the light of current data, PA has an impact on pubertal timing, but the mechanisms behind this phenomenon are not clear. First, excess androgen exposure in PA could lead to increased peripheral conversion of weak adrenal androgens to more potent androgens and estrogens. In a recent longitudinal study on 252 healthy peripubertal girls, serum DHEAS, androstenedione and estrone concentrations rose before thelarche and the increase in serum estradiol concentration (29). The authors did not study the age of menarche in these girls, but suggested that peripheral conversion of adrenal androgens might lead to breast development without gonadotropin activation and might thus slow the tempo from thelarche to menarche. This study setting was different, but our results do not support this hypothesis as menarche was earlier without a significant difference of breast development in the PA girls compared with the controls. Second, adrenal androgens may accelerate gonadotrophin-dependent pubertal development. DHEAS, the most prominent biomarker in PA, is known to act as a neurosteroid (30), and some authors have speculated that adrenal androgens might have some effects on pubertal onset and tempo *via* γ-aminobutyric acid inhibition to the gonadotropin-releasing hormone pulse generator (31). Third, the most probable connection between adrenal and gonadal maturation is insulin–IGF-1 axis, as increased concentrations of IGF-1 and insulin have been associated with both PA (6, 14, 16, 17) and central precocious puberty (32).

Advanced early childhood growth (usually associated with PA) tends to induce greater weight gain, taller stature, and hyperinsulinism in later childhood (33). Hyperinsulinism may contribute to hepatic IGF-1 formation leading to increased IGF-1 concentrations. IGF-1, in turn, may be a key factor in the postnatal adrenal maturation (34) and adrenarche in girls (35). Interestingly, rapid weight gain and growth in early childhood (reflecting good nutritional environment) may induce a childhood endocrine milieu where hyperinsulinism, elevated IGF-1, enhanced adrenal androgen secretion, increased peripheral metabolism of these weak androgens, and lower SHBG concentrations (leading to increased free androgen concentrations) could all promote pulsatile gonadotropin-releasing hormone activity (33). Moreover, some hypothesized etiological factors of PA, including low birth weight (5, 10, 36) and higher animal protein intake in early childhood (37), may promote growth acceleration and earlier pubertal development independently of PA (19, 31). In our own cohort, mostly AGA-born PA girls had early growth acceleration, hyperinsulinism, elevated IGF-1, and decreased SHBG concentrations (14, 20), and as our current study indicates, also earlier pubertal development. Moreover, the PA girls with PP were taller and had more often reached menarche by the age of 12 years than the PA girls without PP indicating a “more severe” phenotype. In addition, earlier menarcheal timing was associated with lower BL SDS and higher prepubertal IGF-1 concentration in all girls of our cohort, and the PA girls with menarche had lower BL SDS, higher prepubertal IGF-1 concentrations, and taller stature at the age of 12 years than the PA girls without menarche. Our results support the hypothesis that early factors in the life of PA girls may contribute to the acceleration of growth and maturation.

Our present study is the first longitudinal study investigating pubertal development and growth of PA children including a healthy control group matched for age and sex. All children were examined at the age of 12 years by one single trained physician, which increases the reliability of the clinical assessment. However, our study has limitations. The age of 12 years does not allow complete evaluation of later pubertal development. The cohort size was relatively small, and 38.4% of the original cohort did not attend the current follow-up visit. Especially for the boys, the number of participants was too small for any strong conclusions. It should be noted that mean cMPH and the percentage of the fathers with early pubertal growth spurt were higher in the PA girls compared with the controls, and maternal menarche was earlier in the PA girls with menarche compared with the PA girls without menarche. Thus, the genetic background may partly explain our observed differences in height and age of menarche between the study groups. Unfortunately, we do not know if the parents themselves have had PA.

In conclusion, the PA girls remained taller and more overweight than the control girls at the age of 12 years, and they presented with earlier menarche. In addition, the PA girls with PP were taller and had earlier menarche at the age of 12 years than the PA girls without PP. The factors predicting earlier menarche in all girls included lower BL and higher prepubertal serum IGF-1 concentration which were stronger determinants than PA or prepubertal concentrations of adrenal androgens. Further longitudinal studies are needed to verify pubertal timing and other long-term consequences of PA.

## Ethics Statement

This study was carried out in accordance with the Declaration of Helsinki. Informed consent was obtained from all participating children and their parents. The protocol was approved by the Research Ethics Committee of the Hospital District of Northern Savo.

## Author Contributions

JJ, RV, and PU conceived the project and designed the study; PU and JL performed collection and handling of the data; JL and JJ analyzed the data; all the authors discussed the data and accepted the final draft; JL wrote the manuscript with contributions from all the authors.

## Conflict of Interest Statement

The authors declare that the research was conducted in the absence of any commercial or financial relationships that could be construed as a potential conflict of interest.

## Abbreviations

AGA, appropriate for gestational age; BL, birth length; BMI, body mass index; cMPH, corrected mid-parental height; DHEAS, dehydroepiandrosterone sulfate; IGF-1, insulin-like growth factor 1; PA, premature adrenarche; PP, premature pubarche; SGA, small for gestational age; SDS, standard deviation score.
